# Associations between anxiety, depression, and risk of suicidal behaviors in Chinese medical college students

**DOI:** 10.3389/fpsyt.2022.1012298

**Published:** 2022-12-02

**Authors:** Jia Li, Yaru Zhang, Bella Siu Man Chan, Sun Nee Tan, Jianping Lu, Xuerong Luo, Yanmei Shen, Xiang Yang Zhang

**Affiliations:** ^1^Department of Applied Psychology, College of Preschool Education, Changsha Normal University, Changsha, Hunan, China; ^2^Department of Psychiatry, and National Clinical Research Center for Mental Disorders, The Second Xiangya Hospital of Central South University, Changsha, Hunan, China; ^3^Department of Educational and Counselling Psychology, and Special Education, The University of British Columbia, Vancouver, BC, Canada; ^4^Djavad Mowafaghian Centre for Brain Health, Faculty of Medicine (Neuroscience), The University of British Columbia, Vancouver, BC, Canada; ^5^Department of Child Psychiatry of Shenzhen Kangning Hospital, Mental Health School, Shenzhen Mental Health Center, Shenzhen University, Shenzhen, China; ^6^CAS Key Laboratory of Mental Health, Institute of Psychology, Chinese Academy of Sciences, Beijing, China

**Keywords:** suicidal behavior, depression, anxiety, medical college students, risk factors, suicide ideation

## Abstract

**Background:**

Previous studies have established a strong association between depression and suicidal behaviors, yet the relationship between anxiety and suicidal behaviors remains unclear. This study examines whether anxiety and depression are independent risk factors for suicidal behaviors in medical college students, and further, whether anxiety may increase the greater risk of suicidal behaviors (SB) in participants with depression.

**Methods:**

This cross-sectional study was conducted among 4,882 medical students. Demographic information, anxiety, and depression data were collected using online questionnaires or through a widely used social media app named WeChat.

**Results:**

Anxiety and depression were independent risk factors for suicidal behaviors, and levels of risk correlated positively with the severity of both anxiety and depressive symptoms. A dose–response relationship was identified between the severity of anxiety and the risk of SB, as well as the severity of depression and SB. Furthermore, anxiety increased the risk of suicidal behaviors in participants with depression, with a dose–response relationship between the severity of anxiety symptoms and the risk of SB.

**Conclusion:**

The findings highlight the importance of screening for anxiety and depressive symptoms in medical college students, as well as reducing anxiety in addition to depressive symptoms in treatment. This study provides valuable data as a reference for clinicians for suicide risk assessments.

## Background

Suicide is one of the leading causes of death throughout the world (1). Over 800,000 people globally die by suicide every year, which accounts for 1.4% of all global deaths (2). Suicide is also regarded as one of the most severe public health problems in China (1). Suicidal behaviors (SB) have a devastating influence on individuals, families, and communities (3, 4), and the prevention of suicide has been recognized as a significant public health challenge. It is therefore important to study SB in order to predict subsequent acts of suicide (3, 4).

Previous studies have suggested that nearly nine out of every ten suicides were associated with mental illnesses (5, 6). Depressive disorder is regarded as the primary mental illness associated with the greatest risk for SB (4, 7). This is affirmed by the Diagnostic and Statistical Manual of Mental Disorders, 4th Edition (DSM-IV), which identifies suicidal ideation as one of the symptoms of major depressive disorder (8). Despite having evidence of a strong association between depression and SB, determining suicide risks among people with depression remains challenging. As SB is considered to be a warning sign of fatal suicide (3), it is crucial to identify risk factors for suicidality among patients with major depression. Furthermore, although previous studies did show a consistent association between the severity of depressive symptoms and suicidality (9, 10), there is a lack of large-scale studies conducted among medical college students examining the association.

Although depression is strongly associated with SB, the role of anxiety symptoms in SB is less clear. Several studies have explored the impact of comorbid anxiety on SB in participants with depression; however, their findings remain inconsistent (7, 11). Recently, several studies showed that comorbid anxiety increased the risk of SB in patients with depression (11, 12). For example, Batterham et al. (11) reported that anxiety symptoms posed an even greater risk overall for SB than symptoms of depression. However, other studies reported different findings. For example, Eikelenboom et al. (7) found that anxiety might be protective against suicidality after adjusting for the severity of depressive symptoms. In their view, anxiety symptoms in depressed patients may stem from psychological fear of their illness, which is the opposite of a wish for death.

Several studies on anxiety, depression, and SB were mostly epidemiologic and clinical studies that examined the relationships between anxiety disorders and suicidality or depressive disorder and suicidality separately (11, 13). The question of whether anxiety is an independent risk factor for suicidality after controlling for comorbid depression has not been adequately addressed (7, 14, 15). Moreover, studies examining the magnitude of anxiety on SB in depression among Chinese populations in general and among Chinese college students in particular are scarce.

To the best of our knowledge, this is the first study to examine the relationship between anxiety and SB in Chinese medical college students with depression. The aims of this study are: first, to explore the associations between the severity of anxiety or depressive symptoms with SB; second, to examine whether anxiety and depression are both independent risk factors for SB after controlling for socio-demographics, severity of anxiety and depressive symptoms and comorbidity, and; third, to investigate the magnitude of anxiety symptoms on SB in a large cohort of participants with depression.

## Materials and methods

### Participants

Students from three medical educational institutions in Hunan, China, were selected as the target population. A cross-sectional design was employed with the convenience sampling method. A total of 80 students from two classes of the Hunan University of Chinese Medicine, 165 students from four classes at Changsha Health Vocational College, and 4,759 students from 50 classes at Yiyang Medical College were chosen to participate. The targeted two medical colleges (Changsha Health Vocational College and Yiyang Medical College) offer 3-year undergraduate programs; students accepted to these kinds of colleges generally have lower academic grades. The Hunan University of Chinese Medicine is a comprehensive medical university with an undergraduate medical program that spans 4 years. Students admitted to this university tend to have competitive academic scores.

A total of 5,004 students were screened between January and March of 2018. All students provided consent and were informed that they could refuse to participate or withdraw at any time. A total of 110 participants declined to participate. Participants who proceeded signed the consent forms, which were approved by the Ethics Committee of the Second Xiangya Hospital at Central South University in Changsha, China; 12 were excluded for incomplete questionnaires or missing data. Therefore, a total of 4,882 participants were enrolled in this study. Of them, 537 were males (11.0%) and 4,345 were females (89.0%). Their ages ranged from 17 to 22 years (mean age: 18.77 ± 1.09 years). College counselors and instructors were given trainings on how to instruct participants to fill out the scales before providing guidance to the respondents. Participants could fill the questionnaires either on a widely used social media app called WeChat (installed on mobile devices) or via computer.

### Measures

#### Socio-economic demographics

The questionnaire included questions about gender, age, nationality, social economic status, whether they smoked or drank, community (urban or rural), parents’ educational levels, and relationships with parents.

#### Suicidal behaviors

Suicidal behavior refers to the thoughts and behaviors related to the intention of committing suicide (16). Suicidal behaviors include suicidal ideation (SI), suicide plans, and suicide attempts (SA) (17). SI refers to passive thoughts about killing oneself or wishing for death. Suicidal plans entail planning and preparation for a suicide attempt. SA referred to any potentially self-harm actions the individual took against themselves, with at least some extent of intention to die (17, 18). Research shows “scales are used in clinical settings to measure the severity of suicidal behavior, and previous research has shown that current suicidal ideation is more easily disclosed via self-report measures than clinical interviews” (19). The latest research shows that “these scales could not discriminate between suicide ideators and suicide attempters” (20). We chose the questions probing suicidal ideations, suicide plans, and SA included: Have you ever had thoughts of committing suicide? Have you ever made a suicide plan? Have you ever tried committing suicide? If any of these questions were answered in the affirmative, additional detailed questions were asked (i.e., frequency of attempts and approaches of SA).

Based on the responses to SB, participants were categorized into two main groups: control and SB groups. Participants exhibiting the specific type of SB were further divided into three subgroups with suicidal ideations, plans, and attempts. Since overlapping occurred among three subtypes of suicidal behavior, those subjects with two or three subtypes of suicidal behavior were included in two or three subgroups.

#### Depression and anxiety

Zung’s Self-Rating Depression Scale (SDS) and Self-Rating Anxiety Scale (SAS) (21, 22) were employed to evaluate symptoms of depression and anxiety over the past 7 days. The SDS and SAS have been broadly employed in studies on depression and anxiety among Chinese populations and are reported to have good psychometric properties (23, 24). The cut-off scores for SDS and SAS are 53 and 50, respectively. Participants with scores higher than 53 on SDS or above 50 on SAS are regarded as having depression and anxiety, respectively (25). The higher scores indicate the greater severity of depression or anxiety. Both the SAS and SDS scales have a 20-item self-report (1 for none or a little of the time, 2 for some of the time, 3 for good part of the time, and 4 for most or all of the time). The scores for items 5, 9, 13, 17, or 19 in SAS and the scores for items 2, 5, 6, 11, 12, 14, 16, 17, 18, or 20 in SDS are reversed (four for none or a little of the time, three for some of the time, two for a good part of the time, and one for most or all of the time). Multiplying the total score by 1.25 provides the standard scores for these two measurements (21, 22).

### Data analysis

The chi-squared test for categorical variables and the analysis of variance (ANOVA) for continuous variables were used to compare the differences in clinical and demographic variables between the groups with different kinds of suicidal behaviors. The binary logistic regression method was applied to calculate the crude odds ratio (OR) and the adjusted OR together with 95% CI for suicidal behaviors (suicide ideations, plans, and attempts) after controlling for the related variables. We used two-tailed *t*-tests to calculate statistical significance with a threshold for *p*-value set at below 0.05 using the SPSS22.

## Results

A total of 4,882 participants in this study were divided into controls (*n* = 3522) and those with SB (*n* = 1360). Further, among those with suicidal behaviors, 1,289 were included in the suicidal ideation subgroup, 371 in the suicide plans subgroup, and 682 in the SA subgroup. Table 1 shows the demographic and clinical data of all the participants. Compared with the control group, individuals with all three subtypes of SB were more likely to have a history of physical disorders, live in urban areas, have a family history of mental disorders, have poor relationships with parents, develop smoking, and drinking habits or have anxiety or depression (all *p* < 0.001).

**TABLE 1:** Demographic characteristics of participants with suicidal behaviors and without suicidal behaviors (controls).

Variables	Controls (*n* = 3522)	Suicidal behaviors (*n* = 1360)	*p*	Suicide ideation (*n* = 1289)	*P^a^*	Suicide plans (*n* = 371)	*P^b^*	Suicide attempts (*n* = 682)	*P^c^*
Age (years), mean (SD)	18.88 ± 1.087	18.57 ± 1.11	0.000***	18.59 ± 1.113	0.000***	18.47 ± 1.123	0.000***	18.48 ± 1.117	0.000***
BMI (kg/m^2^), mean (SD)	20.093 ± 2.4181	20.26 ± 2.53	0.035*	20.278 ± 2.5705	0.021*	19.963 ± 2.4054	0.322	20.117 ± 2.5263	0.816
Males/females	418/3104	119/1241	0.002**	105/1184	0.000***	34/337	0.122	65/617	0.080
District: Rural/Urban	2586/936	934/426	0.001**	885/404	0.001**	228/143	0.000***	457/225	0.001**
Nationality: Others/Han	466/3056	156/1204	0.098	147/1142	0.092	32/339	0.012*	69/613	0.026*
Good relationship with mother	3447 (97.9%)	1276 (93.8%)	0.000***	1210 (93.9%)	0.000***	333 (89.8%)	0.000***	630 (92.4%)	0.000***
Good relationship with father	3402 (96.6%)	1232 (90.6%)	0.000***	1164 (90.3%)	0.000***	315 (84.9%)	0.000***	602 (88.3%)	0.000***
Physical disorder history	177 (5.0%)	149 (11.0%)	0.000***	144 (11.2%)	0.000***	45 (12.1%)	0.000***	85 (12.5%)	0.000***
Mental disorder history	22 (0.6%)	16 (1.2%)	0.049*	15 (1.2%)	0.058	11 (3.0%)	0.000***	15 (2.2%)	0.000***
Family history of mental disorders	50 (1.4%)	44 (3.2%)	0.000***	42 (3.3%)	0.000***	21 (5.7%)	0.000***	28 (4.1%)	0.000***
Smoking	130 (3.7%)	82 (6.0%)	0.000***	78 (6.1%)	0.000***	36 (9.7%)	0.000***	60 (8.8%)	0.000***
Drinking	487 (13.8%)	310 (22.8%)	0.000***	296 (23.0%)	0.000***	101 (27.2%)	0.000***	193 (28.3%)	0.000***
Anxiety	492 (14.0%)	480 (35.3%)	0.000***	460 (35.7%)	0.000***	178 (48.0%)	0.000***	302 (44.3%)	0.000***
Depression	882 (25.0%)	680 (50.0%)	0.000***	654 (50.7%)	0.000***	217 (58.5%)	0.000***	384 (56.3%)	0.000***
**Social economic status**									
Lower than 30,000/year	1478 (42.0%)	577 (42.4%)	0.262	548 (42.5%)	0.384	136 (36.7%)	0.110	284 (41.6%)	0.042*
30,000∼50,000/year	1222 (34.7%)	442 (32.5%)		422 (32.7%)		135 (36.4%)		211 (30.9%)	
More than 50,000/year	822 (23.3%)	341 (25.1%)		319 (24.7%)		100 (27.0%)		187 (27.4%)	
**Father’s education level**									
Junior middle school and below	2284 (64.8%)	874 (64.3%)	0.836	833 (64.6%)		218 (58.8%)	0.016*	436 (63.9%)	0.446
High School or Technical School	901 (25.6%)	359 (26.4%)		335 (26.0%)	0.951	102 (27.5%)		170 (24.9%)	
College or University and above	337 (9.6%)	127 (9.3%)		121 (9.4%)		51 (13.7%)		76 (11.1%)	
**Mother’s education level**									
Junior middle school and below	2591 (73.6%)	965 (71.0%)	0.166	913 (70.8%)	0.166	246 (66.3%)	0.010*	480 (70.4%)	0.216
High School or Technical School	720 (20.4%)	310 (22.8%)		292 (22.7%)		94 (25.3%)		154 (22.6%)	
College or University and above	211 (6.0%)	85 (6.3%)		84 (6.5%)		31 (8.4%)		48 (7.0%)	
**Family type**									
Extended families	1221 (34.7%)	485 (35.7%)	0.000***	460 (35.7%)	0.000***	127 (34.2%)	0.000***	233 (34.2%)	0.001**
Nuclear families	1983 (56.3%)	705 (51.8%)		666 (51.7%)		184 (49.6%)		357 (52.3%)	
Single parent/recombinant family	318 (9.0%)	170 (12.5%)		163 (12.6%)		60 (16.2%)		92 (13.5%)	

BMI, body mass index; *p^a^* was generated by comparison of control group and suicidal ideation subgroup; *p^b^* was generated by comparison of control and suicide plans; *p^c^* was generated by comparison of control and suicidal attempts. *p < 0.05; **p < 0.01; ***p < 0.001.

When compared to participants without anxiety, individuals with anxiety had higher risks of suicidal behaviors (*p* < 0.01). The severity of anxiety symptoms correlated positively with the risk of SB (*p* < 0.01). In the case of suicide plans and suicide attempts, the crude odds ratios (ORs) reached as high as 26.913 and 19.365, respectively (both *p* < 0.001). Anxiety remained an independent risk factor (*p* < 0.001) even after adjustments were made for demographics, smoking, drinking, and severity of depression symptoms. The risk of SB increased with the severity of anxiety symptoms after adjustments (with adjusted ORs of 3.670 for suicide ideation, 7.032 for suicide plans, and 6.164 for suicide attempts). Participants with depression also showed higher risks of SB; the risk of SB increased with the severity of depression symptoms; the crude ORs for suicidal ideation, suicide plans, and SA reached 47.811 (95% CI, 11.243−203.315), 128.571 (95% CI, 29.141−567.256), and 75.302 (95% CI, 17.313−327.522), respectively (all *p* < 0.001). The adjusted ORs remained independent and high even after adjustments were made for demographics, smoking, drinking, and severity of anxiety symptoms (all *p* < 0.001; see Table 2).

**TABLE 2:** Associations between suicidal behaviors with severity of anxiety symptoms and depression symptoms.

	Suicidal ideation		Suicide plans		Suicide attempts	
			
Variable	Crude OR	Adjusted OR	Crude OR	Adjusted OR	Crude OR	Adjusted OR
**Anxiety**						
Without anxiety (SAS ≤ 49)	−	−	−	−	−	−
Mild (50 ≤ SAS ≤ 59)	3.018 (2.571−3.542)***	1.934 (1.605−2.331)***	4.491 (3.506−5.753)***	2.593 (1.916−3.508)***	4.154 (3.423−5.041)***	2.508 (1.987−3.166)***
Moderate (60 ≤ SAS ≤ 69)	5.516 (3.889−7.822)***	2.524 (1.703−3.740)***	12.274 (8.027−18.768)***	3.899 (2.302−6.605)***	8.844 (6.049−12.928)***	3.567 (2.278−5.587)***
Severe (70 ≤ SAS)	11.487 (4.890−26.983)***	3.670 (1.418−9.496)***	26.913 (10.477−69.135)***	7.032 (2.099−23.556)***	19.365 (7.979−46.997)***	6.164 (2.225−17.080)***
**Depression**						
Without depression (SDS ≤ 52)		−	−	−	−	−
Mild (53 ≤ SDS ≤ 62)	2.743 (2.369−3.177)***	2.091 (1.774−2.466)***	3.164 (2.466−4.061)***	1.976 (1.474−2.649)***	3.296 (2.734−3.974)***	2.116 (1.701−2.631)***
Moderate (63 ≤ SDS ≤ 72)	3.833 (3.070−4.785)***	2.328 (1.790−3.029)***	6.696 (4.899−9.152)***	3.196 (2.159−4.733)***	5.122 (3.939−6.660)***	2.438 (1.764−3.370)***
Severe (73 ≤ SDS)	47.811 (11.243−203.315)***	18.526 (4.135−83.003)***	128.571 (29.141−567.256)***	26.636 (5.302−133.811)***	75.302 (17.313−327.522)***	17.194 (3.632−81.393)***

OR, odds ratio. ***p < 0.001.

Table 3 showed the associations between anxiety and SB among participants with depression, as compared with those without anxiety. Those with anxiety showed higher risks of SB in all 3 categories as indicated by increasing values for crude and adjusted ORs. The crude ORs reached 8.568 (95%CI, 3.208−22.880), 16.217 (95%CI, 5.473−48.051), and 13.706 (95%CI, 4.964−37.848) (all *p* < 0.001) for SI, suicide plans, and suicide attempts, respectively. Even after controlling for demographics, smoking, and drinking, participants with anxiety were at higher risk of suicidal behaviors in all 3 categories. The adjusted ORs decreased to some extent but remained significant at 7.538 (95%CI, 2.757−20.606), 12.190 (95%CI, 3.789−39.224), and 11.922 (95%CI, 4.154−34.217) for suicidal ideation, suicide plans, and suicide attempts, respectively (all *p* > 0.01).

**TABLE 3:** Associations between anxiety and suicidal behaviors in participants with depression, with and without adjustment for socio-demographic characteristics, and smoking and drinking habits.

	Suicidal ideation		Suicide plans		Suicide attempts	
			
Variable	Crude OR	Adjusted OR	Crude OR	Adjusted OR	Crude OR	Adjusted OR
**Anxiety**						
Without anxiety (SAS ≤ 49)	−	−	−	−	−	−
Mild (50 ≤ SAS ≤ 59)	1.821 (1.463−2.265)***	1.776 (1.417−2.225)***	2.322 (1.655−3.256)***	2.225 (1.555−3.185)***	2.382 (1.825−3.110)***	2.296 (1.740−3.030)***
Moderate (60 ≤ SAS ≤ 69)	2.902 (1.988−4.237)***	2.470 (1.664−3.669)***	5.424 (3.346−8.792)***	4.319 (2.547−7.324)***	4.335 (2.845−6.606)***	3.734 (2.388−5.840)***
Severe (70 ≤ SAS)	8.568 (3.208−22.880)***	7.538 (2.757−20.606)***	16.217 (5.473−48.051)***	12.190 (3.789−39.224)***	13.706 (4.964−37.848)***	11.922 (4.154−34.217)***

OR, odds ratio. ***p < 0.001.

## Discussion

This study confirmed the association between SB (suicidal ideation, suicide plans, and suicide attempts) and the severity of anxiety and depressive symptoms. Both anxiety symptoms and depressive symptoms were independent risk factors for suicidal behaviors, after controlling for the severity of these symptoms, demographics, and lifestyles (smoking and drinking habits). The severity of anxiety symptoms positively correlated with risks for SB among participants with depression. These findings shed light on the importance of screening for anxiety and depressive symptoms among medical college students as well as highlighting the importance of reducing anxiety symptoms and depressive symptoms to prevent suicide.

Furthermore, this study showed that even after controlling for the severity of depression, smoking, drinking, and demographics, anxiety disorder remained an independent risk factor for SB, although the adjusted OR decreased to some extent. This implies that the risks for suicidal behaviors associated with anxiety symptoms might be increased partly due to anxiety symptoms and partly due to comorbidities such as depression, smoking, and drinking. In addition, we found a dose–response relationship between the severity of anxiety symptoms and the risks of SB: severity of anxiety symptoms correlated positively to risks of SB. The finding that anxiety symptoms were associated with SB is consistent with several previous studies (12, 26–33). Several studies found that after controlling for depression, anxiety symptoms remained an independent risk factor for SB (12, 30, 33). A recent study conducted with a large sample of South Korean adults similarly reported an independent association between anxiety disorders and suicidal ideation and/or suicide attempts after adjustments were made for demographic variables and psychiatric comorbidities (26). Some studies suggested that anxiety might increase the risk of SB by adding more mental burden to the person (34). Other studies recommended that comorbidities should be taken into consideration for psychiatric disorders that often occur concurrently (35). Beck et al. (36) corroborated that hopelessness is the key variable linking depression to suicidal behavior. This finding has direct implications for the therapy of suicidal individuals. Alessandra Costanza highlights that assessment of demoralization may contribute to a more comprehensive suicide risk detection by the findings of a systematic review (37). Rodriguez and Kendall (38) suggested that the increased risks of SB among anxiety-disordered individuals might be related to intolerance of distress and emotional dysregulation. Evidence has shown that participants with anxiety display poorer tolerance of distress (39) and more emotion dysregulation (40, 41). Such individuals find it very difficult to tolerate and modulate negative emotions and may be more inclined to think about suicide as a way to gain relief from their distress or as a method of escape (38).

However, other studies reached alternative conclusions regarding the correlation between anxiety and SB. Some studies suggested that the correlation could just be a by-product of comorbid depression (7). Moreover, a specific part of the anxiety, namely, the somatic anxiety, might be protective against suicidality (7). Several explanations might be responsible for the inconsistency in the conclusions. First, the inconsistency may be in part caused by sample differences; participants mentioned in previous studies range from community members (26) to college students (33) to clinical patients (42). Second, the conclusions may have been inconsistent because the relationships between anxiety and suicidal behaviors vary according to subtypes of anxiety disorders. For example, a number of studies that did not find an independent association between anxiety and SB (43, 44) only examined the severity and symptomatology of generalized anxiety. In contrast, many studies that affirmed the positive association examined a wide range of anxiety disorders or employed broader measures of anxiety symptoms (45–48). The potentiality that specific anxiety disorders have varying relationships to suicidal behaviors therefore warrants further investigation. Third, the discrepant conclusions may reflect the different relationships that anxiety disorders have with the wide spectrum of SB (e.g., suicidal ideations, suicide plans, or suicide attempts). It is important to note that not all previous research works on anxiety and suicidality have addressed these components separately. Perhaps anxiety disorders are specific predictors of the SI, but not of the capability or intention to engage in suicide attempts (38).

This study revealed a positive correlation between depression and suicidal behaviors, which is consistent with previous studies (28, 49, 50). Depression was shown to be an independent risk factor for SB after adjustments were made for demographic variables and comorbidities, which is also consistent with previous studies (7, 51). The strong association between depression and suicidal behaviors revealed in this study highlights the importance of paying attention to depressive symptoms in medical college students. As only 0.6% of the participants reported a history of mental disorder, this seemingly low prevalence of self-identified depression may be a result of stigma. Studies have suggested that medical students refrain from disclosing their depression with school counselors as they worry that they would be less respected, seen to have less adequate coping skills, and considered to be less capable of handling their responsibilities (52). As a result, medical students with depression do not get diagnosed or treated, which could in turn exacerbate their symptoms and increase their risk for suicidality. Thus, there is a need to develop a positive mental health environment in medical schools in China to provide medical students with easy access to mental health resources and treatment options. In this study, participants scoring high on suicidal ideation were recommended to seek help from school counselors. The recommendation is specified on the webpage after finishing the questionnaire.

Several studies reported that anxiety increased the risk of SB when comorbid with depression (12, 31–33, 53, 54). The finding from this study contributes to the question of how anxiety impacts SB in patients with depression using data from a large sample size, cross-sectional study. Our findings are consistent with results from the studies mentioned above and revealed a dose–response relationship between the severity of anxiety symptoms and the risks of SB in depression. Some studies suggested that concurrent anxiety disorders exacerbate symptoms of depression, which manifests into even more psychological distress, poorer physical functioning, and poorer social functioning (55)– all of which may account for the increased the risk of SB. In addition, patients with comorbid anxiety had lower scores on scales that measure their quality of life (56) and were reported to have a longer duration of depression (57) and more emotional distress (57), posing a negative influence on the risk of suicidal behaviors. However, several other studies suggested inconsistent conclusions (7, 57, 58): some studies found that anxiety actually protected individuals from SB (7, 58). The conflicting findings have several explanations. First, the inconsistency regarding findings on the relation between anxiety and suicidality could result from heterogeneities in methodology. For example, Li et al. (59) suggested that gender ratios, sample sizes, response rates, and differences in screening tools explained almost half of the inter-study variations. Second, anxiety disorders include Panic Disorder, Agoraphobia, Obsessive Compulsive Disorder, Posttraumatic Stress Disorder, Acute Stress Disorder, and Generalized Anxiety disorder, as well as specific and social phobias. The effect of anxiety on SB in depression varied greatly with different kinds of anxiety disorders (7). Third, as previous studies suggested a serial of mediation from anxiety to SB through degree of depressive symptoms (34), the effect of anxiety on risks of SB may also differ in response to different levels of severity of depressive symptoms. It is also possible that depressive symptoms have a moderating effect on the relationship between anxiety and SB. In addition, Beck’s work questioned whether hopelessness was more predictive than depression with regard to suicide risk (36). And assessment of demoralization may contribute to a more comprehensive suicide risk detection by the findings of a systematic review (37). In other words, the relationship between anxiety and risks for SB may change depending on the severity of depressive symptoms. Future studies need to be conducted in order to investigate this possibility.

This study has several limitations. First, self-report assessment scales were utilized to measure symptoms of anxiety, depression, and SB. Participants’ stigmas toward mental illness may have led them to under-report their symptoms (59). Second, the study used a cross-sectional study design, which means the data were collected from one time point and so were insufficient to establish any causal relationships between anxiety symptoms, depression symptoms, and suicidal behaviors. Future research should employ a longitudinal design in order to examine the direct causality of SB with other related variables. Third, most of the participants were females. Future studies should include an equal sample size of both genders to increase representativeness of population. Finally, the sample consists of data collected from three medical colleges, most of them were from Yiyang medical school, it may not be representative of Chinese college students in general and is possibly not representative of the adult population in China, future studies should enroll a larger range of samples to improve the representativeness. Therefore, more future studies are required so that repeated, reproducible results from varying populations can confidently inform us of the interactions and relations, whether causal or correlation, among SB, anxiety, and depression symptoms (60). It is noteworthy to emphasize that future studies that recruit large sample sizes from different sample populations would be most beneficial.

## Conclusion

In summary, this study found that SB was very common among medical students, besides, anxiety, and depression were independent risk factors for SB. Participants with severe anxiety and depression symptoms were at much high risk of SB. This indicates the importance of screening for anxiety and depression among medical college students in order to promote mental health wellbeing and prevent SB. This study also found that anxiety was associated with an increased risk of SB among individuals with depression, and, the risk of SB was increased with the severity of anxiety symptoms. All findings lead to important clinical implications. First, SB was very common among medical students; it is imperative to screen for SB as well as anxiety and depressive symptoms among medical college students and to better prevent suicide. Second, the study also provides valuable data for healthcare professionals to refer to when assessing the risks of suicide for patients with anxiety, depression, and comorbid depression and anxiety.

## Data availability statement

The original contributions presented in this study are included in the article/supplementary material, further inquiries can be directed to the corresponding authors.

## Ethics statement

The research protocol was approved by the Ethics Committee of the Second Xiangya Hospital, Central South University, China. All procedures performed in studies involving human participants were in accordance with the ethical standards of the institutional research committee and with the 1964 Helsinki declaration and its later amendments or comparable ethical standards.

## Author contributions

XL, XZ, JL, and YS were responsible for the study design. JL, YZ, and JPL were responsible for recruiting the participants. BS and ST were involved in statistical analysis. JL and JPL were involved in manuscript preparation and drafting the manuscript. XL, BS, and ST were involved in editing and revising the manuscript. YS and XZ were responsible for the critical revision of the manuscript. All authors have contributed to the manuscript and had given approval for the submission of the manuscript.

## Abbreviations

DSM-IV: Diagnostic and Statistical Manual of Mental Disorders, 4th Edition
SDS: Zung’s Self-Rating Depression Scale
SAS: Self-Rating Anxiety Scale
OR: odds ratio
BMI: Body Mass Index.
